# Role of S100A3 in human colorectal cancer and the anticancer effect of cantharidinate

**DOI:** 10.3892/etm.2013.1344

**Published:** 2013-10-14

**Authors:** BIN LIU, WEN-YI SUN, CHEN-YANG ZHI, TIAN-CHENG LU, HAI-MEI GAO, JIAN-HUA ZHOU, WEI-QUN YAN, HAI-CHENG GAO

**Affiliations:** 1 Department of General Surgery, Jilin University Second Hospital, Changchun, Jilin 130041, P.R. China; 2 Department of Clinical Pharmacy and Pharmaceutical Management, School of Pharmaceutical Sciences, Jilin University, Changchun, Jilin 130021, P.R. China; 3 Changchun University of Traditional Chinese Medicine, Changchun, Jilin 130118, P.R. China; 4 School of Life Sciences, Jilin Agricultural University, Changchun, Jilin 130118, P.R. China; 5 Colon Department, Affiliated Hospital of Changchun University of Traditional Chinese Medicine, Changchun, Jilin 130021, P.R. China

**Keywords:** colorectal cancer, S100A3, cantharidinate

## Abstract

Colorectal cancer (CRC) is a leading cause of cancer-related mortality. The early diagnosis and treatment of CRC is the key to improving the survival of patients who may benefit from adjuvant chemotherapy. In the present study, the protein expression of S100A3 was observed in a cohort of 20 patients with cancer, which indicated that S100A3 activation was involved in tumorigenesis. In addition, the anticancer activity of cantharidinate was investigated using immunohistochemistry and quantitative polymerase chain reaction (qPCR) analysis. The protein expression of S100A3 was observed to increase by 2.4-fold in human CRC cells compared with the expression level in normal control cells (P<0.01). Cantharidinate inhibited the protein and gene expression of S100A3 in UCT-116 human CRC cells *in vitro*. These results suggested that S100A3 is important in human CRC. Cantharidinate has the potential to be considered as a novel adjuvant drug for controlling the expression of S100A3 in human CRC as it exhibits preventive effects.

## Introduction

Colorectal cancer (CRC) is a cancer that develops from uncontrolled cell growth in the colon or rectum (parts of the large intestine), or in the appendix (1). Genetic analysis has shown that colon and rectal tumors are essentially the same type of cancer (2). The symptoms of CRC typically include rectal bleeding and anemia, which may occur with weight loss and changes in bowel habits (3). The majority of the cases of CRC occur due to lifestyle and increasing age; only a minority of cases are associated with underlying genetic disorders (4). The disease typically starts in the lining of the bowel and, if left untreated, may grow into the muscle layers underneath and then through the bowel wall (5). Cases of CRC that are confined within the wall of the colon are often curable with surgery, while cancer that has spread widely around the body is usually incurable. In such instances, disease management focuses on extending the life of the patient using chemotherapy and improving the patient's quality of life (6). CRC is the third most frequently diagnosed type of cancer in males and the second most frequently diagnosed type of cancer in females, and was estimated to account for >1.2 million new cancer cases and 608,700 mortalities in 2008 (7). At present, there is a focus on chemotherapy for tumors (8). Despite the fact that considerable progress has been made in recent years, the pathogenesis and treatment of CRC remain unclear.

S100A3 is a matricellular protein, which is expressed in numerous tissues and cell types (9). The S100A3 protein is a protein that in humans is encoded by the S100A3 gene. The protein encoded by the S100A3 gene is a member of the S100 family of proteins containing two EF-hand calcium-binding motifs (10–13). Over the last decade it has become increasingly apparent that S100A3 is an important mediator, although it is unclear whether S100A3 is important in CRC and whether it is possible to inhibit S100A3 with drug treatment. At present, fluorouracil is one of the standard chemotherapeutic drugs used in the treatment of CRC (14). In the past decade, the treatment options for CRC have expanded and include additional chemotherapeutic agents and targeted therapies (cetuximab, panitumumab and bevacizumab) (15). The proper use of these therapies has had a major impact on the prognoses of patients (16).

In recent years, data concerning the treatment of cancer with traditional Chinese medicine have had a considerable influence with regard to the identification of specific molecular markers and pathway aberrations that may guide treatment decisions (17). However, it has not yet been elucidated whether traditional Chinese medicine is able to inhibit the expression of S100A3 and prevent the symptoms of CRC. Cantharidin (also its acid form cantharidinate) has been used in traditional Chinese medicine (18,19). Cantharidinate induces cell cycle arrest and triggers apoptosis in various types of tumor cells, including hepatoma, myeloma, oral buccal carcinoma, leukemia, gastric cancer, TSGH-8301 human bladder carcinoma, Colo205 CRC, A549 human lung cancer and intestinal epithelial cells (20–23).

In the present study, we investigated whether S100A3 is important in CRC and whether cantharidinate may be used to inhibit the expression of S100A3.

## Materials and methods

### Patients and tissue specimens

Twenty patients, comprising 12 males and 8 females, with an average age of 68.25 years (range, 21–87 years) were included in this study. Human CRC tissue specimens were obtained by surgical resection from May 2011 to June 2012 in the Jilin University Second Hospital (Changchun, China). The study was approved by the Ethics Committee of Jilin University Second Hospital (no. 2012-43) and patient consent was obtained. Tissue microarrays (TMAs) were constructed. The histological grade of the tumor and its site (colon or sigmoid colon) were recorded.

### Histopathological examination

The specimens and cells were examined under a light microscope (Eclipse TE-2000-U, equipped with an attached digital camera SXM1200F, Nikon, Tokyo, Japan) following hematoxylin and eosin (H&E) staining.

### Immunohistochemical staining

Paraffin-embedded slices, measuring 4 μm in thickness, were probed with anti-human S100A3 monoclonal antibody (Sigma, St. Louis, MO, USA; 1:300 diluted for use) at 4°C overnight. The sections were then immersed in 0.3% H_2_O_2_ in absolute methanol for 15 min to block endogenous peroxidase. The color was developed using the chromagen 3,3′-diaminobenzidine (DAB) with ABC immunohistochemistry kits from Beijing Biosynthesis Biotechnology Co., Ltd. (Beijing, China). The slices were subsequently counterstained with hematoxylin, mounted on glass coverslips and sealed with neutral resin.

### UCT-116 cell culture and treatment

UCT-116 human CRC cell lines were donated by the Jilin University Institute of Regenerative Medicine. UCT-116 cells were routinely cultured in Dulbecco's modified Eagle medium (DMEM; Gibco-BRL, Invitrogen Life Technologies, Gaithersburg, MD, USA) supplemented with 20% fetal bovine serum (FBS) and 50 U/ml antibiotics under the conditions of 5% CO_2_ at 37°C. Following trypsinization to passage the cells, the cells were incubated in DMEM with 0.5% FBS for 24 h and then treated with 2.5 μmol/l cantharidinate (verified by Professor B. Liu, Jilin University Second Hospital) or 2.5 μmol/l fluorouracil in DMEM with 0.5% FBS, respectively, or were left untreated as control cells for 48 h.

### Quantitative polymerase chain reaction (qPCR) analysis

Total RNA (mRNA) was extracted from colorectal cancer and adjacent non-tumorous tissues using TRIzol^®^ reagent (Invitrogen Life Technologies, Carlsbad, CA, USA), in accordance with the manufacturer's instructions. Total RNA (1 μg) was reverse transcribed to complementary DNA (cDNA) with oligo (dT) primers. The sense and antisense primers for S100A3 were designed according to the mRNA sequence (GenBank accession no. NM-002960.1) and synthesized by Shanghai Sangon Biological Engineering Co. Ltd. (Shanghai, China). Amplified PCR fragments spanning different exons were used to prevent the amplification of contaminated genomic DNA. The primer sequences of S100A3 were as follows: sense, 5′-GACCATCTGGTTCAGGTTCC-3′ and antisense, 5′-ACATTCCCGAAACTCAGTCG-3′. The PCR products were 200 bp in length. The housekeeping gene reduced glyceraldehyde-phosphate dehydrogenase (GAPDH) was used as an internal control, with the primer sequences as follows: sense, 5′-CCAGGTGGTCTCCTCTGACTT-3′ and antisense, 5′-GTTGCTGTAGCCAAATTCGTTGT-3′.

### Statistical analysis

The statistical analysis of the data was performed using SPSS statistical software version 11 for Windows (SPSS, Inc., Chicago, IL, USA). All data are presented as the mean ± standard error of the mean. Statistical comparisons were conducted using the Student's t-test; P<0.05 was considered to indicate a statistically significant difference.

## Results

### Clinicopathological features and patient outcome

The present study was performed on a TMA constructed from the surgical resection samples of patients with CRC with a range of grades of differentiation. The samples were collected as part of a trial comparing the expression of S100A3 in carcinoma and control tissues. The demographics of the patients are shown in Table I, along with the clinicopathological features of the colon and sigmoid and the grades of differentiation. There was an incidence of 8.3% for grades IIB, IIC, IIIB and IIIA CRC, respectively, in males. In females, the incidence of grades IIB and IIC CRC was zero, and the incidence of grades IIIB and IIIA was 25.0 and 12.5%, respectively. The incidence of cancer in the colon and sigmoid colon was 75 and 25% in males, and 50 and 50% in females, respectively.

### H&E of human CRC tissues

**Figure 1:**
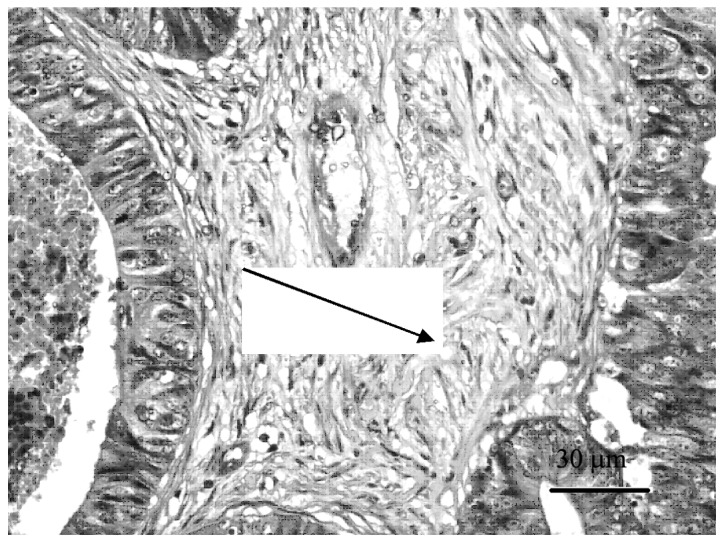
Pathological examination of human colorectal cancer (CRC) from a 60-year-old male using hematoxylin and eosin (H&E) staining (magnification, ×400). The histological analysis shows a highly differentiated CRC with dense arrangements of atypical glands and cancer cells with large and deeply-stained nuclei, arranged in a disorderly manner. Bar indicates 30 μm. The arrow indicates the region of interest.

**Figure 2:**
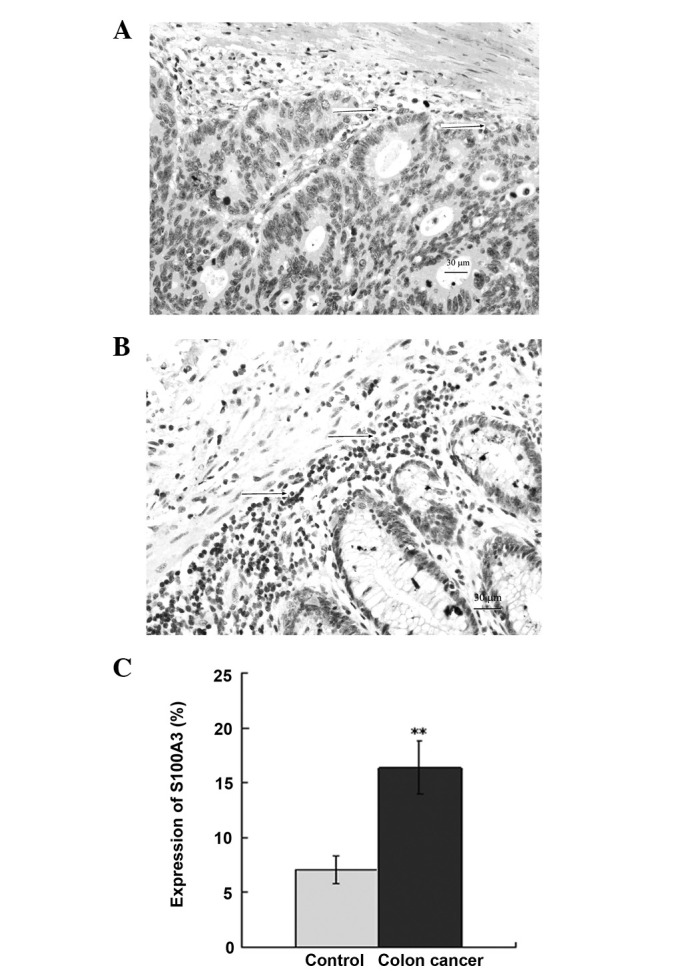
Immunohistochemical staining of: (A) peripheral normal control tissues and (B) colorectal cancer (CRC) tissues with increased S100A3 expression (magnification, ×400). Arrows indicate the region of interest. Positive S100A3 immunohistochemical staining was brownish yellow. (C) The semiquantified level of S100A3 protein in the CRC tissues was increased significantly compared with that in the normal control tissues (**P<0.01, Student's t-test).

Following conventional H&E staining, the CRC TMAs were observed to have representative histological structures when viewed under a microscope (Fig. 1). The immunohistochemical staining of the microarray samples was representative (Fig. 2).

### Immunohistochemical staining of S100A3 in human CRC tissues

Immunohistochemical staining was used to assess the protein expression of S100A3 in human CRC tissues. The results showed that S100A3 was expressed in the membrane and cytoplasm in the normal tissue of the patients with CRC (Fig. 2A). The expression of S100A3 increased notably in the CRC tissues (Fig. 2B), with the expression predominantly in the tumor and tumor interstitial regions. There was a significant difference between the expression of S100A3 in the CRC and normal tissues (P<0.01; Fig. 2C).

### H&E staining of UCT-116 cells

**Figure 3:**
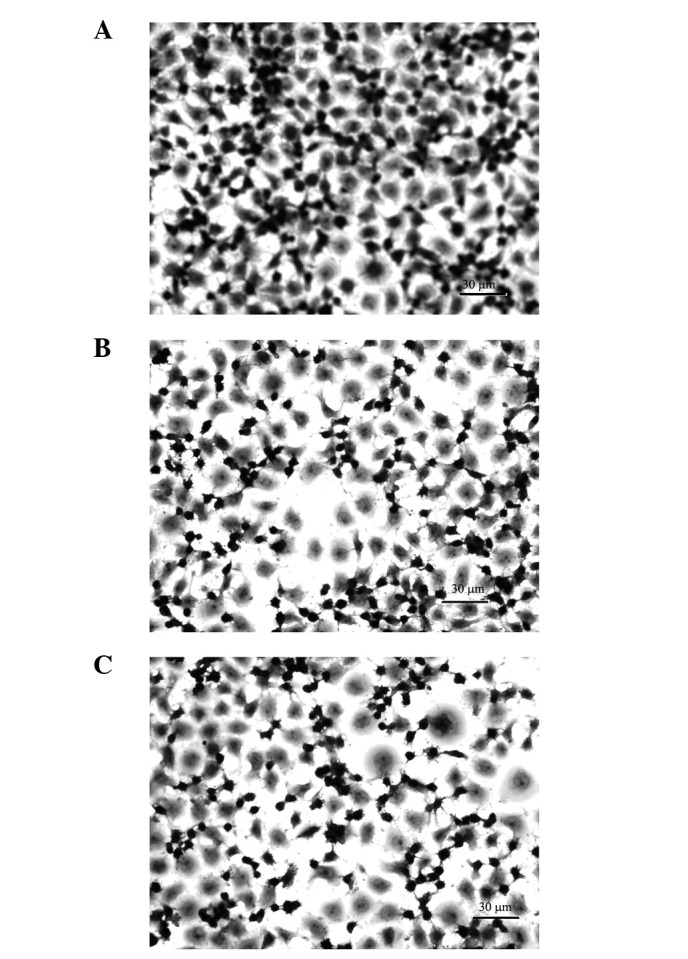
Hematoxylin and eosin (H&E) staining of UCT-116 human colorectal cancer (CRC) cells: (A) control, (B) fluorouracil-treated and (C) cantharidinate-treated (magnification, ×400). Fluorouracil and cantharidinate markedly reduced the cancer cell count compared with that of the control cells.

The pathological changes in the different groups of UCT-116 human CRC cells are shown in Fig. 3. The quantity of UCT-116 cells decreased 48 h subsequent to the application of fluorouracil and cantharidinate (Fig. 3B and C). This result suggested that cantharidinate may reduce the time required for chemotherapy and subsequently decrease the risk of the treatment for human CRC.

### Immunohistochemical staining detects S100A3 expression in UCT-116 cells

**Figure 4:**
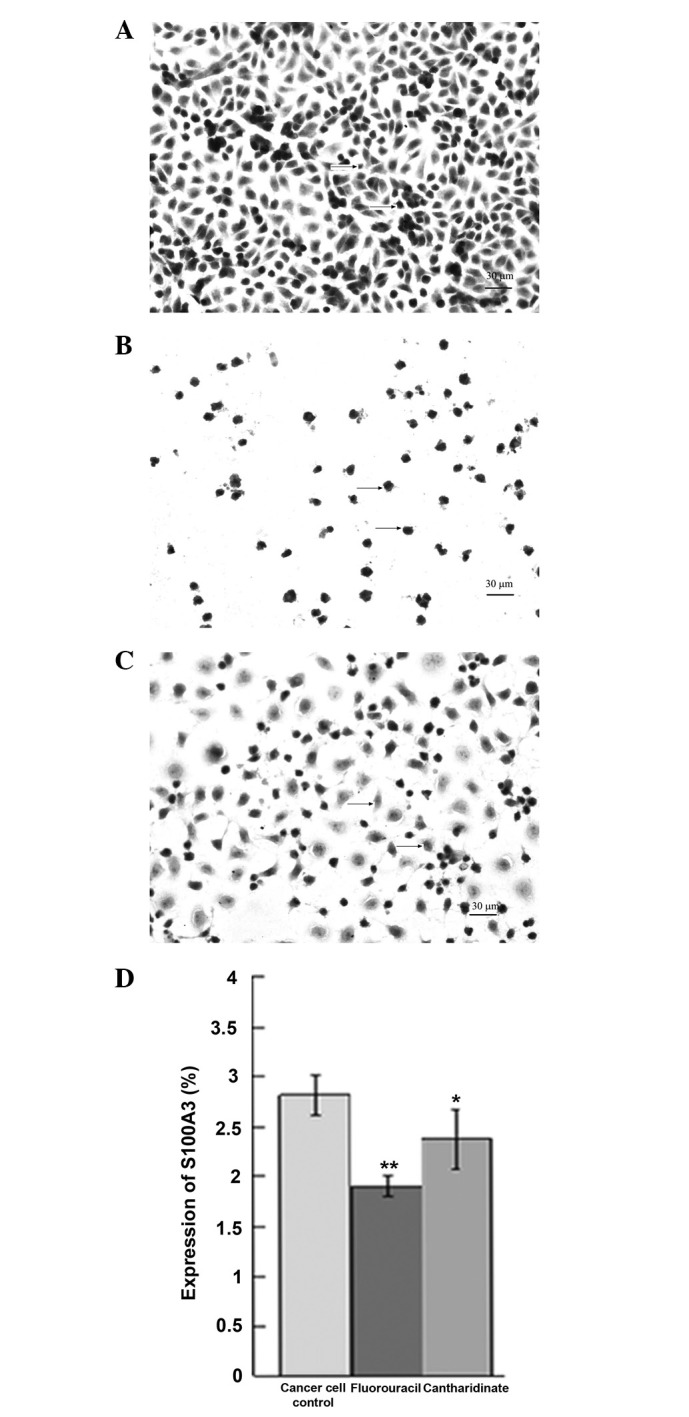
Immunohistochemical staining of S100A3 in UCT-116 human colorectal cancer (CRC) cells: (A) control, (B) fluorouracil-treated and (C) cantharidinate-treated. Arrows indicate the region of interest (magnification, ×400). Positive S100A3 immunohistochemical staining was brownish yellow. (D) The semiquantified level of S100A3 protein shows that fluorouracil and cantharidinate treatments reduced the expression of S100A3. **P<0.01, *P<0.05 compared with cancer cell control.

Fig. 4 shows the S100A3 expression in UCT-116 cells, as observed using immunohistochemical staining. A high level of S100A3 protein was detected in the untreated UCT-116 cell controls (Fig. 4A and D). The cantharidinate and fluorouracil treatments each reduced the level of S100A3 in the UCT-116 cells significantly (P<0.05; Fig. 4B–D). The protein expression of S100A3 increased by 2.4-fold in human CRC cells compared with the expression level in normal control cells (P<0.01). These results suggest that cantharidinate is able to inhibit the expression of S100A3 and, therefore, may have the ability to block tumor growth.

### qPCR analysis of result

**Figure 5:**
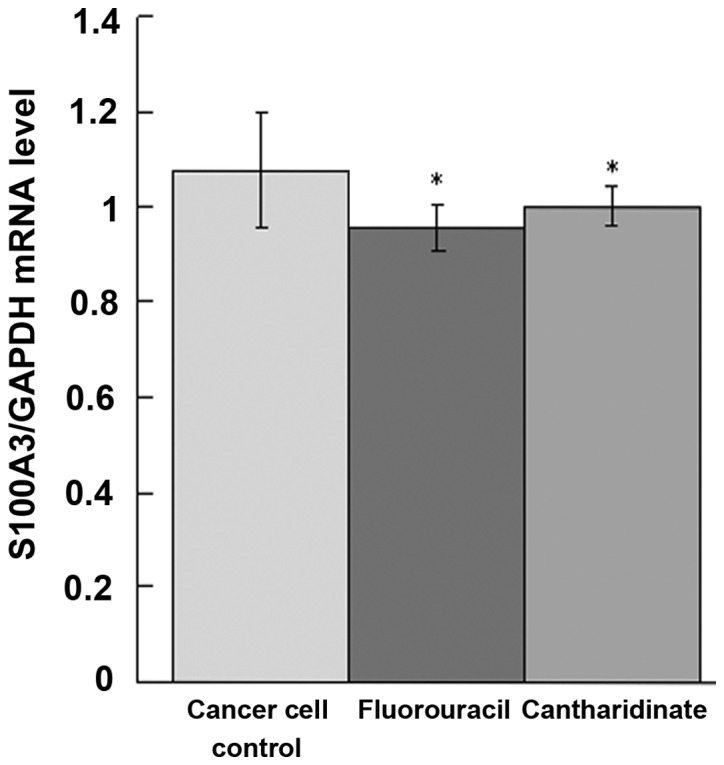
Quantitative polymerase chain reaction (qPCR) was used to detect the expression of S100A3 mRNA in control, fluorouracil-treated and cantharidinate-treated UCT-116 human colorectal cancer (CRC) cells. Fluorouracil and cantharidinate treatments decreased the S100A3 mRNA levels to 0.81- and 0.88-fold, respectively. *P<0.05 compared with cancer cell control.

To determine whether the S100A3 mRNA level in the UCT-116 cells changed following the application of cantharidinate, qPCR analysis was performed. As shown in Fig. 5, the expression of S100A3 mRNA in the cantharidinate group decreased to 0.88-fold that of the untreated cancer cell controls (P<0.05). The level of S100A3 in the cantharidinate group was similar to that in the fluorouracil group. This result suggested that the ability of cantharidinate to kill tumor cells may be due to its ability to inhibit S100A3 mRNA expression (Fig. 5).

## Discussion

CRC is the third most frequently diagnosed cancer in the world. It is more common in developed countries (24), with ~60% of cases being diagnosed in the developed world (25). However, the pathogenesis on CRC remains unclear. The prevention of genetic mutations and treatment of mutant genes is of critical significance. Studies have demonstrated that S100A3 belongs to a family of structurally and functionally associated proteins that are widely distributed in tumors. There has been a surge in studies that have produced results indicating that the dysregulated expression and function of S100A3 contributes to pathological conditions, such as cancer metastasis, celiac disease and diseases associated with defective assembly (10–13).

It is unclear whether S100A3 is significant in CRC. The present study demonstrated that the level of S100A3 was increased in the process of tumor occurrence and progression, and that S100A3 expression in human CRC was inhibited by cantharidinate. A desirable property of an anticancer drug is the ability to induce the death of tumor cells with few side-effects on normal cells (16,26). The present study demonstrated that cantharidinate has inhibitory activity against S100A3 in human CRC. The effects of cantharidinate were similar to those of fluorouracil. Cantharidinate was able to inhibit the proliferation of human CRC cells with an IC_50_ value of 2.5 μM and exhibited little cytotoxicity in normal cells (data not shown). The present study demonstrated that cantharidinate reduced the mRNA and protein expression of S100A3 in human CRC cells. To the best of our knowledge, this is the first study that has shown that the mRNA and protein expression levels of S100A3 are downregulated by cantharidinate (18–23).

In conclusion, S100A3 is important in human CRC. Cantharidinate is able to inhibit the expression of S100A3 and may be considered as a novel additional drug that may be used to control the expression of S100A3 in human CRC and the growth of human CRC.

## Figures and Tables

**Table I:** Clinicopathological features of the cohort of patients with colorectal cancer (n=20).

Variable	Male (n=12)	Female (n=8)
Age (years)		
Mean	63.50	73.38
Range	21–80	63–87
Minimum (%)	21 (12.5)	63 (25.0)
Maximum (%)	80 (12.5)	87 (37.5)
Grade of differentiation (n)		
IIB (%)	1 (8.3)	0 (0)
IIC (%)	1 (8.3)	0 (0)
IIIB (%)	1 (8.3)	2 (25.0)
IIIA (%)	1 (8.3)	1 (12.5)
Colon (%)	9 (75.0)	4 (50.0)
Sigmoid (%)	3 (25.0)	4 (50.0)
